# Mood Symptoms and Chronic Fatigue Syndrome Due to Relapsing-Remitting Multiple Sclerosis Are Associated with Immune Activation and Aberrations in the Erythron

**DOI:** 10.3390/brainsci13071073

**Published:** 2023-07-14

**Authors:** Abbas F. Almulla, Al-Karrar Kais Abdul Jaleel, Ali Abbas Abo Algon, Chavit Tunvirachaisakul, Hayder K. Hassoun, Hussein K. Al-Hakeim, Michael Maes

**Affiliations:** 1 Department of Psychiatry, Faculty of Medicine, Chulalongkorn University, Bangkok 10330, Thailand; abbass.chem.almulla1991@gmail.com (A.F.A.); chavit.tun@gmail.com (C.T.); 2 Medical Laboratory Technology Department, College of Medical Technology, The Islamic University, Najaf 54001, Iraq; 3 Medical Laboratory Department, Kufa Institute, Al-Furat Al-Awsat Technical University, Najaf 54001, Iraq; alkkais50@gmail.com; 4 Iraqi Education Ministry, Najaf 54001, Iraq; ali.aboalgon@gmail.com; 5 Cognitive Impairment and Dementia Research Unit, Faculty of Medicine, Chulalongkorn University, Bangkok 10330, Thailand; 6 Faculty of Medicine, University of Kufa, Kufa 54002, Iraq; drhayder67@hotmail.com; 7 Department of Chemistry, College of Science, University of Kufa, Kufa 54002, Iraq; headm2010@yahoo.com; 8 Department of Psychiatry, Medical University of Plovdiv, 4002 Plovdiv, Bulgaria; 9 Research Institute, Medical University of Plovdiv, 4002 Plovdiv, Bulgaria; 10 University of Electronic Science and Technology of China, Chengdu 611731, China

**Keywords:** cytokines, neuroinflammation, neuroimmune, chronic fatigue syndrome, depression, mood disorders

## Abstract

Background: Multiple sclerosis (MS) is a chronic autoimmune and neuroinflammatory disease of the central nervous system characterized by peripheral activation of immune-inflammatory pathways which culminate in neurotoxicity causing demyelination of central neurons. Nonetheless, the pathophysiology of relapsing-remitting MS (RRMS)-related chronic fatigue, depression, anxiety, cognitive impairments, and autonomic disturbances is not well understood. Objectives: The current study aims to delineate whether the remitted phase of RRMS is accompanied by activated immune-inflammatory pathways and if the latter, coupled with erythron variables, explain the chronic fatigue and mood symptoms due to RRMS. Material and Methods: We recruited 63 MS patients, 55 in the remitted phase of RRMS and 8 with secondary progressive MS, and 30 healthy controls and assessed erythron variables, and used a bio-plex assay to measure 27 serum cytokines. Results: A significant proportion of the MS patients (46%) displayed activation of the immune-inflammatory response (IRS) and compensatory immune response (CIRS) systems, and T helper (Th)1 and Th17 cytokine profiles. Remitted RRMS patients showed increased chronic fatigue, depression, anxiety, physiosomatic, autonomic, and insomnia scores, which could partly be explained by M1 macrophage, Th1, Th-17, growth factor, and CIRS activation, as well as aberrations in the erythron including lowered hematocrit and hemoglobin levels. Conclusions: Around 50% of remitted RRMS patients show activation of immune-inflammatory pathways in association with mood and chronic-fatigue-like symptoms. IRS and CIRS activation as well as the aberrations in the erythron are new drug targets to treat chronic fatigue and affective symptoms due to MS.

## 1. Introduction

Multiple sclerosis (MS) is a chronic disease characterized by a cluster of symptoms comprising ataxia, numbness, visual disturbances, loss of balance, unexplained pain, chronic fatigue, muscle spasms, bladder malfunction, depression, cognitive impairments, and progressing disabilities [1,2,3]. MS shows a higher prevalence in high-income populations (North America 140/100.000 and Europe 108/100.000 compared with 2.2 and 2.1/100,000 in East Asia and sub-Saharan Africa, respectively) [4]. In Iraq, the female/male ratio of MS is 2.18:1 [5].

MS can be classified into relapsing-remitting MS (RRMS), secondary progressive MS (SPMS), primary progressive MS (PPMS), and clinically isolated syndromes (CIS) [6]. While RRMS is the most prevalent MS phenotype, some of these patients develop SPMS accompanied by progressive deterioration leading to loss of neurological functions [7]. Two mechanisms explain MS-associated disabilities as evaluated by extended disability scale scores (EDSS) [8], namely, the gradual accumulation of dysfunctions resulting from poor recovery from acute relapses (denoted as relapse-associated worsening), and progression independent of relapse activity [9,10]. MS disabilities are cumulative, and the speed of their accumulation can be assessed by the multiple sclerosis severity scale (MSSS) [9].

MS is a chronic autoimmune and neuroinflammatory disease of the central nervous system (CNS) and spinal cord resulting in demyelination of neurons [11,12]. Autoimmunity, neuroinflammation, oxidative stress damage, excitotoxicity, axonal and neuronal damage, demyelination, neurodegeneration, glial scar formation, remyelination, and impairments of metabolic and mitochondrial metabolism are strongly involved in the acute relapses and progression of MS [13,14,15,16]. Increased levels of 13 cerebrospinal fluid (CSF) and 21 blood cytokines were reported in people with MS [17]. For example, tumor necrosis factor (TNF)-α, CXCL-8, interleukin (IL)-15, IL-12p40, and CXCL13 were found to be elevated in CSF and blood [17]. 

High burdens of chronic fatigue, depression, and anxiety are reported in MS and may impair the patient’s quality of life and interfere with the remission potential and thus prognosis of the illness [18,19,20,21]. The prevalence of chronic fatigue, depression, and anxiety in MS patients is between 75–90%, 27.01%, and 35.19%, respectively [22,23]. Depression and anxiety may be more prevalent in PMS than in RRMS [23], while there are no differences in chronic fatigue between both MS subtypes [24]. Activated immune-inflammatory and oxidative and nitrosative stress (O&NS) pathways may partially explain the onset of mood symptoms and chronic fatigue in MS [2,18,19]. Elevated concentrations of M1 macrophage and T helper (Th)1 cytokines along with high levels of reactive oxygen species (ROS), lipid peroxidation, and nitric oxide (NO) play a role in depression due to MS [2]. Moreover, a significant correlation was found between the severity of depression due to MS and increased expression of TNF-α and IFN-γ genes [25,26]. A number of studies have indicated that chronic fatigue syndrome, major depressive disorder (MDD), and generalized anxiety disorder are associated with activated immune-inflammatory and O&NS pathways [27,28,29,30]. For example, clinical depression is accompanied by increased levels of interleukin (IL)-1β, IL-6, IL-8, IL-17, IFN-γ, and TNF-α [27].

In general, it is more difficult to interpret solitary cytokine levels and, therefore, it is preferable to describe cytokine findings in terms of profiles, including M1 macrophage, Th1, Th2, Th17, T regulatory (Treg), growth factor, and T-cell growth factor profiles and to combine these further into composites reflecting activation of the immune-inflammatory response system (IRS) versus the compensatory immunoregulatory system (CIRS) [27]. The latter system comprises immunoregulatory and anti-inflammatory mechanisms such as Th2 and Treg cytokines (e.g., IL-4 and IL-10) or soluble receptors (e.g., soluble IL-1 receptor antagonist (sIL-1RA) and sIL-2R), which tend to downregulate the IRS and counteract an overzealous inflammatory response [27]. In this respect, MS is associated with the activation of Th1, Th17, Th9, and Th22 profiles, whilst Tregs also play a key role [31,32,33]. Clinical depression is accompanied by activated M1, Th1, Th17, T-cell growth, and IRS and CIRS profiles, although during acute episodes the IRS/CIRS ratio is increased [27]. Nevertheless, there are no data on these profiles in depression, anxiety, and chronic fatigue due to RRMS.

Aberrations in the erythron may be involved in the pathophysiology of MS and depression. For example, Hon et al. found lower levels of hemoglobin (Hb) in MS patients compared with healthy controls along with a significant inverse correlation between red blood cell (RBC) count and EDSS scores [34]. Moreover, the RBC count is significantly decreased in patients with RRMS compared to other types of MS and healthy controls [35]. Depressed patients have significantly lowered RBCs and hematocrit (Hct) and Hb levels compared with healthy controls, whereas red cell distribution width (RDW) and reticulocytes are significantly elevated, suggesting inflammation-associated anemia [36,37,38]. However, there are no data on the association between erythron features and IRS activation, chronic fatigue, and mood symptoms due to RRMS.

Hence, the aim of the current study is to delineate the IRS/CIRS and erythron profiles of chronic fatigue and affective symptoms due to MS and RRMS.

## 2. Material and Methods

### 2.1. Participants

In the present case-control study, 63 patients with MS were recruited at the Neuroscience Center of Alsader Medical City in Al-Najaf province, Iraq, from September 2021 to March 2022. A senior neurologist diagnosed patients using the McDonald Criteria 2010 [39]. The RRMS patients (n = 55) had suffered two or more episodes and were in the remitted phase of the illness, defined as a recovery phase post-relapse with symptom disappearance and no disease progression. Eight patients suffered from SPMS. We also recruited 30 healthy individuals, either staff, friends of staff, or medical workers as a control group from the same catchment area as the patients. All the individuals were free of (lifetime and current) axis-1 neuropsychiatric disorders including a major depressive episode, schizophrenia, bipolar disorder, psycho-organic disorders, substance-use disorders (except nicotine dependence), medical disorders including chronic fatigue syndrome, diabetes mellitus, cardiovascular, thyroid, renal, liver, gastrointestinal, oncologic disorders, and (auto)immune, other neuroinflammatory, and neurodegenerative diseases, including psoriasis, chronic obstructive pulmonary disease, inflammatory bowel disease, Parkinson’s disease, and Alzheimer’s disease.

MS patients or their parents/legal guardians and controls gave written signed consent before inclusion in the study. The approval to conduct the current study was obtained from the institutional ethics board of the College of Medical Technology, The Islamic University of Najaf, Iraq (doc. no 11/2021). In this study, we followed Iraqi and foreign ethics and privacy rules based on the guidelines of the World Medical Association Declaration of Helsinki, the Belmont Report, the Council for International Organizations of Medical Sciences guidelines, and the International Conference on Harmonization of Good Clinical Practice. Our IRB adheres to the International Guideline for Human Research Safety (ICH-GCP).

### 2.2. Clinical Assessment

A senior psychiatrist evaluated sociodemographic, neuropsychiatric, and clinical data through a semi-structured interview. The EDSS [8] was used for the clinical evaluation of disabilities, while the MSSS [9] was used to evaluate the progression of disability over time reflecting the severity of MS. Activities of daily living (ADL) were determined using the Arabic-translated index of ADL [40]. In addition, the Hamilton Depression Rating Scale (HAMD) [41], Hamilton Anxiety Rating Scale (HAMA) [42], the Beck Depression Inventory, and the Fibro-Fatigue Scale (FF) [43] were used to determine the severity of depressive, anxiety and fibromyalgia-fatigue symptoms, respectively. We asked participants who took part in the study to rate their depression, anxiety, and FF complaints over the three months preceding the study. Based on our previous publications [44,45,46], we computed several symptom subdomain scores as z unit-based composite scores using the z scores of different items, namely: (a) pure depressive symptoms computed as the sum of depressed mood + feelings of guilt + suicidal ideation + loss of interest (HAMA) + sadness (FF), depressed mood (HAMA) + sadness + discouraged about the future + feeling a failure + dissatisfaction + feeling guilty + disappointed in oneself + critical of oneself + suicidal ideation + crying + loss of interest + difficulty with decisions + look unattractive + work inhibition (BDI); (b) pure anxiety (pure HAMA) as the sum of anxious mood + tension + fears + anxiety behavior at interview; (c) pure physiosomatic symptoms as a z unit-based composite score based on the sum (z scores) of anxiety somatic + gastrointestinal + genitourinary + hypochondriasis somatic sensory + cardiovascular + gastrointestinal (GIS) + genitourinary + autonomic symptoms + respiratory symptoms (HAMA symptoms) + muscle pain + muscle tension + fatigue + autonomic + gastrointestinal + headache + malaise (FF scale) + fatigue (BDI) + anxiety somatic + somatic gastrointestinal + general somatic + genital symptoms + hypochondriasis (HAMD); (d) fatigue as the sum of fatigue (FF) + fatigue (BDI); (e) autonomic symptoms as autonomic symptoms (FF and HAMA) and sleep disorders as sleep disorders (FF) + early + middle + late insomnia (HAMD) + insomnia (HAMA) + sleep disturbance (BDI). We divided participant’s body weight in kilograms by the square of their height in meters squared to obtain the body mass index (BMI).

### 2.3. Biochemical Assays

In the early morning (8:00–11:00 a.m.), 5 mL of venous blood was withdrawn from all participants by disposal syringe and divided into EDTA and serum tubes. A complete blood count (CBC) was performed on all subjects utilizing EDTA whole blood. Serum was obtained after centrifugation of the blood at 35,000 rpm, then stored as small aliquots using Eppendorf tubes and frozen at −80 °C until thawed for biomarker assays. We used a hematology analyzer (HumaCount 30) provided by the Human Company (Wiesbaden, Germany) to perform CBC. A Bio-plex Pro™ Human Chemokine Assays (Bio-Rad Laboratories, Inc. (Hercules, CA, USA) was employed to analyze a panel of cytokines and chemokines, including IL-1β, IL-1ra, IL-2, IL-4, IL-5, IL-6, IL-7, IL-8, IL-9, IL-10, IL-12(p70), IL-13, IL-15, IL-17, Eotaxin, fibroblast growth factor (FGF) basic, granulocyte colony-stimulating factor (G-CSF), granulocyte-macrophage colony-stimulating factor (GM-CSF), IFN-γ, IP-10, MCP-1(MCAF), macrophage inflammatory protein-1 (MIP-1)-α, PDGF-bb, MIP-1β, regulated upon activation, normal T-cell expressed and secreted (RANTES), TNF-α, and vascular endothelial growth factor (VEGFs). The procedure was as follows: (a) using sample diluent (HB), the serums were diluted (1:4), (b) 50 microliters of the diluted samples were mixed with 50 µL of microparticle cocktail (containing cytokines/chemokines per well of a 96-well plate provided by the manufacturer) and incubated for 1 h at room temperature while shaking at 850 rpm, (c) the plate was rinsed three times, then 50 µL of diluted Streptavidin-PE was added to each well and the plate was incubated (with shaking at 850 rpm) for 10 min at room temperature. (d) 125 µL of assay buffer was added, and the wells were shaken at room temperature (850 rpm) for 30 s before being read using the Bio-Plex^®^ 200 System (Bio-Rad Laboratories, Inc., California, USA). We utilized the immunofluorescence (IF) values of the cytokines/chemokines in the data analyses [47,48]. Cytokines/chemokines which showed >35% out-of-range concentration levels were not entered in the analysis, although the IF values were used to compute z unit-based composite scores (see below) [47,48]. Consequently, we did not include IL-2, IL-5, IL-7, IL-15, GM-CSF, and VEGF in the regression analyses when using separate cytokines/chemokines. The intra-assay CV values for all analytes were <11.0%. In the current study, the primary outcomes were various immune profiles, including the M1 macrophage profile computed as z IL-1β  +  z IL-6  +  z TNF-α  +  z CXCL8  +  z CCL3  +  z sIL-RA; Th1 as z IL-2  +  z IL-12  +  z IFN (interferon)-γ; Th2: z IL-4  +  z IL-9  +  z IL-13; Th17: z IL-6  +  z IL-17; and T-cell growth (all factors that promote T-cell growth): z IL-4  +  z IL-7  +  z IL-9  +  z IL-12  +  z IL-15  +  z GM-CSF [27,47]. Neurotoxicity was conceptualized as a composite score comprising neurotoxic cytokines/chemokines: z IL-1β  +  z TNF-α  +  z IL-6  +  z IL-2  +  z IFN-γ  +  z IL-17  +  z CCL11  +  z CXCL10  +  z CCL3  +  z CCL5  +  z CCL2 [48]. Based on a recent paper [49] and the available cytokines, we also computed a Th17-axis index as z IL-6 + z TNF-α + z IL-17. The Th1/Th2 ratio was computed as zTh1–zTh2. 

### 2.4. Statistical Analysis

We employed SPSS (Chicago, IL 60606 United States) Windows version 28 to perform all statistical analyses. The comparisons between category-based variables were accomplished by analysis of contingency tables (Chi-square tests). The differences between study groups in terms of continuous variables were determined by analysis of variance (ANOVA). In addition, we utilized Quade Nonparametric Analysis of Covariance (ANCOVA) to examine differences in the non-normally distributed immune activation variables while covarying for CIRS values to estimate the levels of immune activation above and beyond that of CIRS activation. Pairwise comparisons among group means were analyzed (at *p* < 0.05) to define differences between the three study groups. Furthermore, corrections of multiple comparisons were made utilizing the false discovery rate (FDR) *p*-value [50]. Multivariate regression analyses were conducted to examine the best immunological and erythron profile predictors of the disability and symptom subdomain scores while allowing for the effects of age, sex, and BMI. In addition, we also used a stepwise automated approach with *p*-values of 0.05 for model entry and 0.1 for model elimination. We estimated the model statistics (F, df, and *p*-values), total variance explained (R^2^), and standardized beta coefficients with t statistics and exact *p*-values for each predictor. Furthermore, the variance inflation factor (VIF) and tolerance were examined to check collinearity and multicollinearity issues. The White and modified Breusch–Pagan homoscedasticity tests were used to check for heteroskedasticity. To define the best cytokine and erythron profiles that predict disability and subdomain scores while allowing for the effects of confounders, we employed automatic linear modeling analyses with the best subsets and the overfit prevention criterion. To normalize the distribution of the data, we used logarithmic or rank inverse-normal transformations. Principal component analysis (PCA) was performed as a feature reduction method to extract validated principal components (PCs) from a set of interconnected data. PCs should comply with prespecified model fit criteria, including explained variance > 0.5, all loadings > 0.7, Kaiser–Meyer–Olkin (KMO) index > 0.6, and an adequate anti-image correlation matrix. The significance was assessed using two-tailed tests with a *p*-value of 0.05. A priori calculation of the sample size using G*Power 3.1.9.4 showed that using an effect size of 0.2 at *p* = 005 (two-tailed) and power = 0.08, the estimated sample size should be at least 65.

## 3. Results

### 3.1. Sociodemographic, Clinical, and Blood Parameters of the Study Groups 

Table 1 displays the sociodemographic characteristics, clinical symptoms, and blood parameters of the MS patients and healthy controls. There were no differences in age, marital status, BMI, and proportion of smokers between MS patients and controls. There were more females in the MS sample and more unemployed participants. This table also shows the measurements of MSSS, EDSS, and ADL scores in patients and controls. The latter data show that our patients have mild disabilities and symptoms. We were able to extract one PC from the EDSS, MSSS, and ADL scores (KMO = 0.617, Bartlett’d test of sphericity χ^2^ = 126.03, df = 3, *p* < 0.001; AVE = 73.57, all loadings > 0.745). This PC reflects an integrated index of disabilities due to MS (labeled as PC_disabilities).

Since the erythron may be influenced by sex, we performed factorial GLM analyses with diagnosis and sex as factors. Age and BMI did not have any effect on these analyses, while RBC counts, Hb, Hct, and PC_RBCs were significantly lower in females than in males (all *p* < 0.001). We were able to extract one PC from the RBC, Hct, and Hb data (KMO = 0.684, Bartlett’d test of sphericity χ^2^ = 130.81, df = 3, *p* < 0.001; AVE = 77.59, all loadings > 0.856), which reflects cell counts, labeled PC_RBCs. We were able to extract one PC from the MCH, MCHC, and RDW-CV data (KMO = 0.680, Bartlett’d test of sphericity χ^2^ = 89.045, df = 3, *p* < 0.001; AVE = 73.62, all loadings > 0.825), labeled as PC_RBCindices. Table 1 shows that there were no significant differences in RBCs, Hct, Hb, and PC_RBCs between patients and controls. MCV and MCH were significantly lower in patients than in controls, while RDW was significantly increased in patients, and there was a trend towards lowered PC_RBCindices. Since sex may influence the erythron parameters, we checked consequent regression analyses by introducing sex as a covariate.

All neuropsychiatric rating scale subdomain scores were significantly higher in patients than in controls. We were able to extract one latent vector from the pure depression, anxiety, and FF subdomain scores (KMO = 0.852, Bartlett’s test of sphericity χ^2^ = 278.00, df = 15, *p* < 0.001; AVE = 62.24%, all loadings > 0.678). This first PC extracted from the symptom domains is labeled PC_PP (PC_psychopathology). There was a significant association between PC_disabilities and PC_PP and all symptom domain scores (all r > 0.515, *p* < 0.001, n = 93) in the combined group of patients and controls. In the total group combined (r = 0.686, *p* < 0.001, n = 93) and in the restricted group of patients (r = 0.339, *p* = 0.006, n = 63), there were significant associations between PC_disabilities and PC_PP.

#### 3.1.1. Assessment of Immune Profiles

Since we observed that a large number of the patients, but not all, showed increased IRS levels, we have performed clustering analyses to discover whether a valid cluster of patients with signs of IRS activation may be retrieved. Therefore, we conducted a two-step cluster analysis with patients versus healthy controls as the categorical variable and M1, Th1, Th2, Th17, and Treg phenotypes as clustering variables. With a good silhouette measure of cohesion and separation of 0.62, three clusters were generated. These groups are healthy controls (n = 30) and MS patients with high (n = 29) and low IRS (n = 34) activation scores. Table 2 shows the different immune profiles of the patients and healthy controls and the results of Quade’s nonparametric ANCOVA with pairwise comparisons among group means. In addition to confounders, we also used CIRS as a covariate to examine the activation of different IRS phenotypes above and beyond CIRS activity. The current study’s results indicate that patients with high MS have significantly increased activity of M1, Th1, Th17, T-cell growth, and growth factors compared with the low MS group and healthy controls. However, no significant differences were found between MS patients with low MS and healthy controls. As such, two valid clusters are formed, one with a comparable immune profile as detected in controls, and a cluster with increased IRS activation. In addition, patients with high IRS activity showed increased neurotoxicity scores. Increased Th2 and CIRS responses were established in the low MS group but not in the high MS group. There were no differences in Th1/Th2 ratio between patients and controls and there were no differences in any of the immune profiles between RRMS and SPMS.

The patients were treated with either IFN-β or betapherone (n = 40), natalizumab (n = 15), or other drugs (fingolimod: n = 4, hydrocortisone: n = 2, methylprednisolone: n = 2). Even without FDR *p*-correction, there were no significant effects of any of the drugs on the immune variables and no differences in immune profiles between these three treatment groups. Moreover, there was no significant association between the three medication groups and the cluster-analysis-derived MS groups (χ2 = 4.20, df = 2, *p* = 0.134).

#### 3.1.2. Prediction of Disabilities and Severity of MS by Immune Biomarkers

**Figure 1:**
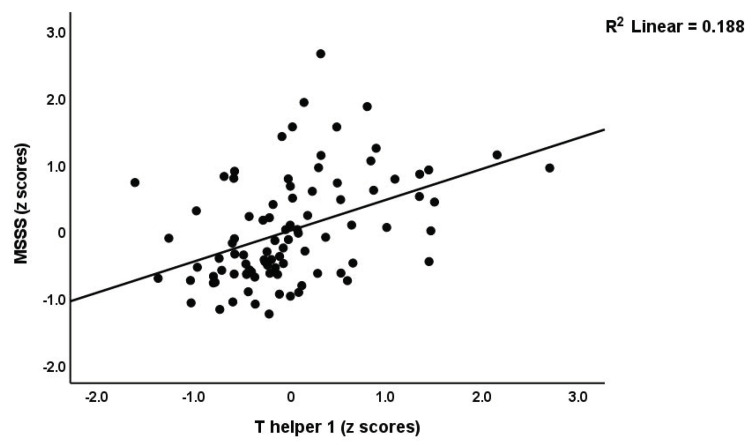
Partial regression of the multiple sclerosis severity scale (MSSS) scores on the T helper 1 profile.

Regressions #1 and #2 were performed on all participants (Table 3) and show that a significant part of the variance (42.2%) in PC_disabilities could be explained by Th1, CIRS, and Th17-axis. Nevertheless, within the selected group of MS patients, no such correlations could be established. Regression #2 displays that T-cell growth, CIRS, and Th1 could explain a significant amount of the variance (53.3%) in MSSS, with all predictors being positively associated. Figure 1 shows the partial regression of the MSSS score on the Th1 profile. In the selected group of patients with MS, 17.8% of the variance in the MSSS score was predicted by Th1 and CIRS functions combined. In the combined study group, we found significant inverse correlations between PC_RBCs and PC_disabilities (r = −0.264, *p* = 0.011, n = 93) and PC_MSSS (r = −0.251, *p* = 0.015, n = 93). Nevertheless, these effects and PC_RBCindices were not significant in the multiple regression analyses shown in Table 3.

#### 3.1.3. Prediction of Neuropsychiatric (NP) Symptoms by Erythron Variables and Immune Indices

**Figure 2:**
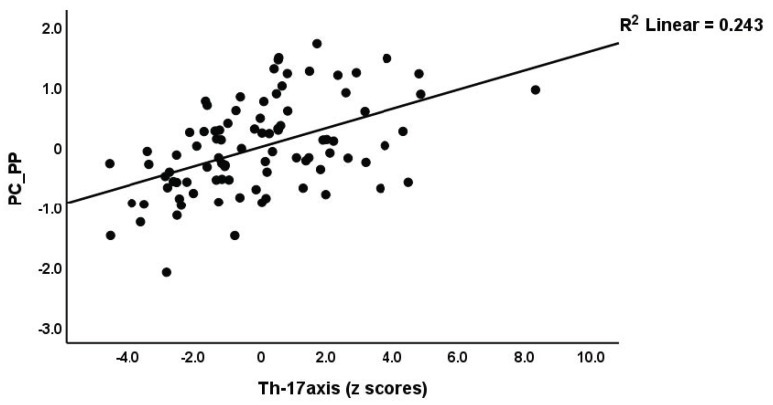
Partial regression of the first principal component score extracted from all psychopathology (PP) scores on the T helper 17-axis profile (*p* < 0.01).

**Figure 3:**
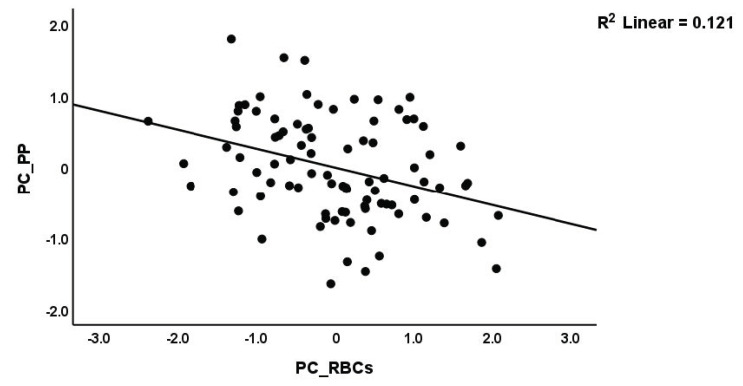
Partial regression of the first principal component score extracted from all psychopathology (PP) scores on the first PC extracted from the number of red blood cells, hematocrit, and hemoglobin (PC_RBCs) (*p* < 0.01).

**Figure 4:**
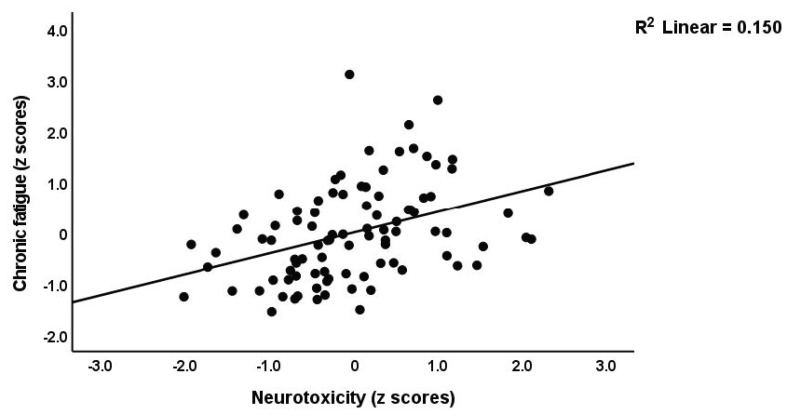
Partial regression of chronic fatigue symptoms due to multiple sclerosis on neuroimmune-toxicity (*p* < 0.01).

We performed regression analysis with neuropsychiatric symptoms as the dependent variables and PC_RBCs, PC_RBCindices, and immune profiles as explanatory variables (Table 4). Regression #1 shows that a large part of the variance (50.6%) in PC_PP was explained by Th17-axis, white blood cells count (WBCs), CIRS (all positively associated), and PC_RBCs (inversely associated). The forced entry of sex into this regression analysis showed that sex was not significant (t = −0.52, *p* = 0.607) and that PC_RBC remained significant (t = −2.55, *p* = 0.013). Figure 2 and Figure 3 show the partial regression of the PC_PP on the Th17-axis and PC_RBCs, respectively. In addition, the forced entry of MSSS into the analysis (regression #2) shows that 56.3% of the variance in PC_PP was explained by MSSS, WBCs, T-cell growth (positively associated), and sex. Regression #3 shows that when we included patients only, 17.1% of the variance in PC_PP could be explained by WBCs (positive association) and PC_RBCs (inverse association). These associations remained significant after entering sex, which was not significant (t = 0.52, *p* = 0.603). Regressions #4 to #9 show the regressions of the various subdomain scores on the cytokines and RBC profiles, while allowing for the effects of demographic data. The highest effect size was established for pure physiosomatic symptoms which showed that 50.3% of its variance could be explained by the Th17-axis, WBCs, CIRS (all positively associated), and sex (regression #4). PC_RBCs showed a significant effect on all subdomains except the pure physiosomatic, fatigue, and sleep domains. These effects remained significant even after the forced entry of sex into the regression analyses. A larger part of the variance in fatigue (41.7%) was explained by neurotoxicity, sex, and CIRS. Figure 4 shows the partial regression of fatigue on neurotoxicity.

### 3.2. Results of Automatic Linear Modeling Analyses with Overfit Prevention

Table 5 shows the results of automatic linear modeling (best subsets with overfit prevention criterion) with PC_disabilities, PC_PP, and the subdomain scores as dependent variables, and the serum erythron variables, WBCs, cytokines, chemokines, and growth factors as explanatory variables, while allowing for age, sex, BMI, and smoking. Regression #1 shows that the best predictors of PC_disabilities are IFN-γ, IL-17, and sIL-RA (all positively associated), which together explain 45.9% of the variance in PC_disabilities. Regression #2 indicated that 47.4% of the variance in PC_PP score could be explained by WBCs, IL-10, IL-6 (positively), and PC_RBCs (inversely). The best predictors of the subdomain scores were WBCs and different cytokines including IFN-γ, IL-4, IL-9, IL-10, IL-13, TNF-α, MIP1A, MCP1 (all positively), and PC_RBCs and sIL-1RA (both inversely).

## 4. Discussion

### 4.1. Immune Profiles in the Remitted Phase

The first major finding of the present research is that a significant proportion (50.9%) of remitted RRMS patients show activated IRS and CIRS, M1, Th1, Th17, and T-cell growth factor profiles. Moreover, we established that a combination of Th1 and Th17-axis activation with increased IFN-γ and IL-17 and increased CIRS indicators are associated with MS-related disabilities. As such, a proportion of the remitted patients shows an ongoing generalized immune-inflammatory process with increased neurotoxic capacity, indicating that the pathophysiological factors of RRMS are still active despite treatments with, for example, betapherone (IFN-1β) and natalizumab.

The current findings extend previous results reporting that significant elevations of central and peripheral Th-1/Th-2 cytokines, including TNF-α, IL-10, sIL-RA, and other components of the IRS/CIRS are observed in RRMS patients who are in the remission phase [51,52]. Kallaur et al. detected an imbalance between Th1, Th2, and Th17 cytokines in remitted MS patients [53]. Hollifield et al. reported a significantly lowered TGF-β and IFN-γ, IL-1β, T-cell mitogen (PHA), and myelin basic protein in peripheral blood mononuclear cells (PBMC) of remitted MS patients [54]. It was suggested that the active phase differs from the remission phase in terms of abnormal cytokine profiles [18]. For example, during the relapse phase of RRMS (defined as new or worsening symptoms persisting 24 h or more, appearing at least 30 days post previous relapse), TGF-β1 was significantly decreased while this cytokine was increased in the remission phase, indicating upregulated CIRS and Treg activities [55,56,57,58]. Moreover, Th17 is upregulated along with a significant increase in IFN-γ-expressing Th17 lymphocytes in the CSF of MS patients during the relapse phase [59,60]. Furthermore, increased Th17 is frequently observed in RRMS [61], and IL-17A and IL-17F are associated with the number of relapses [62].

The ongoing IRS/CIRS response with upregulated M1, Th1, and Th17-axis profiles and IFN-γ production during the remitted phase may contribute to the breakdown of the blood–brain barrier (BBB) and chronic neurotoxicity [63], and, thus, demyelination. CNS neuroinflammation may be aggravated by the migration of peripheral inflammatory mediators across the damaged brain endothelial cells of the BBB [64]. Moreover, immune activation, either IRS or CIRS, may cause viral reactivation, which may play a role in MS. For example, increased CIRS activity may lead to immunosuppression, which may reactivate latent viral infections. Following relapses, female, but not male, patients may display reactivation of Epstein–Barr virus (EBV) in B lymphocytes [65]. The higher relapse rate in RRMS is accompanied by increased expression of some human endogenous retroviruses (HERVs) [66]. Reactivation of MS-associated retrovirus (MSRV) from the HERV-W family may drive IRS activation via Toll-Like Receptor activation, although MSRV reactivation may also result from IRS activation [67]. It should be added that increased production of IFN-γ and IL-17, which is associated with increased disabilities during the remitted phase, may play a role in autoimmunity via different mechanisms [68].

Subsequent investigations ought to explore whether the cohort of patients who have achieved remission and display heightened immune reactions are predisposed to experiencing new relapses or manifesting an accelerated relapse or transitioning into SPMS. It is suggested that a drug target for preventing new relapses should involve a broader activation of the immune system, encompassing M1, Th1, IFN-γ, Th17-axis, IRS, and CIRS profiles, rather than targeting a singular aspect of the immune system.

### 4.2. Immune Profiles and Depression, Anxiety, and Physiosomatic Symptoms due to MS

The second major finding of the current study is that fatigue, depression, and anxiety scores are significantly higher in remitted RRMS and SPMS patients as compared with healthy controls and that MS-related disabilities during the remitted phase are strongly associated with chronic fatigue, depression, and anxiety, physiosomatic and autonomic symptoms, and insomnia. Since we assessed HAMD, HAMA, and FF scales over the three months preceding the study, the results indicate that small increases in disabilities are accompanied by increased chronic fatigue and chronic affective symptoms. During relapses, depressive symptoms are more prominent than in the remission phase [69], although other reports show non-significant associations between depressive symptoms and different clinical phases [70].

Importantly, chronic fatigue and affective symptoms during the remission RRMS phase are largely predicted by the Th17-axis, and increased CIRS and WBC numbers, along with increased IL-10, IL-6, IL-9, IL-13, IL-4, and IFN-γ. As such, increased activation of the Th17-axis and CIRS during the remitted phase of RRMS may drive chronic fatigue and affective symptoms due to MS. Previous reports showed that IL-6 is positively associated with depressive symptoms due to RRMS [2,71,72] and PMS [73]. Chronic fatigue syndrome is considered to be an immune-inflammatory disease with many pathophysiological similarities to multiple sclerosis, including a variety of immunological and neurological abnormalities, such as immune activation, immunosuppression, mitochondrial dysfunctions, elevated synthesis of nuclear factor-κB, and autoimmunity, as well as increased oxidative damage and decreased antioxidant capacity [16]. Likewise, depression is characterized by activated IRS, CIRS, and oxidative stress pathways [27,74,75,76].

Previously, it was reported that increased IL-17, IL-6, and TNF-α (the components of the Th17-axis in the current study) contribute to major depressive disorder and chronic fatigue symptoms [16,27,33,49,77,78,79,80,81,82]. Interestingly, the first paper that reported increased TNF-α in major depression, found that the serum levels of this cytokine were higher in major depression than in MS [83]. Morris and Maes concluded that, in MS, immune activation, including activation of the Th17-axis components, may drive chronic fatigue and physiosomatic symptoms [16].

The neurotoxic effects of the Th17-axis components, which play a key role in MS and fatigue and affective symptoms due to MS, arise from their role in peripheral inflammation, gut barrier and BBB breakdown, microglial activation, neuroinflammation, and neurotoxicity to CNS circuits [49,77,81,84,85]. Activated immune-inflammatory pathways and consequent aberrations in brain structure and functions may render MS patients prone to develop mood symptoms and chronic fatigue syndrome [16,73,86]. It should be added that the increased fatigue and affective symptoms during acute relapses [87,88] may be ascribed to activated immune-inflammatory and nitro-oxidative stress pathways [19,75,81,89,90,91].

### 4.3. Fatigue and PP due to MS and the Erythron

The third major finding of the present study is that chronic fatigue and affective symptoms due to MS, but not MS per se, are associated with deficits in the erythron, namely, lowered numbers of RBCs, Hct, and Hb, whereas alterations in RBC indices have less impact. Thus, aberrations in RBC indices (especially increased RDW) are a hallmark of MS, but not chronic fatigue and affective symptoms due to MS. Our findings extend previous results showing that the erythron profile of MS patients indicates altered RDW values [92] and that, in MS, there is a significant association between high RDW and EDSS scores [93]. Other hemorheological features of MS comprise an increased aggregation of RBCs due to increased peripheral inflammation [92]. RBC numbers, Hb, and Hct may differ significantly between RRMS and SPMS and are lower in RRMS compared to normal controls [35]. Furthermore, major depression is associated with abnormal erythron parameters including decreased RBCs, Hct, and Hb, probably as a consequence of the chronic mild inflammatory response during that illness [36,37]. In chronic fatigue syndrome, RBCs are less deformable and show lower membrane fluidity and zeta-surface charge compared with RBCs of healthy controls [94]. It is common knowledge that anemia and iron deficiency may cause chronic fatigue symptoms.

Our results suggest that aberrations in the erythron in MS may contribute to the pathophysiology of chronic fatigue and affective symptoms due to MS. First, disorders in the erythron resulting from activated IRS may induce the hypoxia-inducible factor (HIF) pathway leading to hypoxic damage [95] as repeatedly reported in fatigue, depression, and anxiety [96]. Second, RBCs have important antioxidant defenses including superoxide dismutase, catalase, and glutathione peroxidase activities [97], whilst hemoglobin may also serve as an antioxidant [97]. Increased lipid peroxidation, which accompanies IRS activation [98], may suppress the antioxidant defenses in the RBCs and change the morphometric features of RBCs and functions of Hb, whilst these changes may be attenuated by the administration of antioxidants [99]. Moreover, the antioxidant capacity of RBCs is reduced in MS leading to increased oxidative stress [92], which plays a key role in chronic fatigue syndrome and affective symptoms [16,98]. Thus, disturbances in RBCs and their antioxidant capacity coupled with high peripheral oxidative stress and changes in blood rheology may increase the vulnerability to oxidative damage and contribute to ischaemic tissue damage [92] and, therefore, to chronic fatigue and affective symptoms.

## 5. Limitations

First, the results would have been more informative if we had assessed biomarkers of oxidative stress including lipid peroxidation and RBC superoxide dismutase, autoimmune biomarkers, and neurotoxic tryptophan catabolites, which are induced during immune activation. Second, although this study was performed on a smaller study sample, the sample size was estimated a priori, based on a power of 0.8. Moreover, post hoc computation of the achieved power, given the computed effect and sample sizes and alpha, show that the power of the regression analyses shown in Table 4 ranges between 0.92 and 1.0. Third, future research should examine the differences in immune and erythron profiles between the remission and acute relapse phases of RRMS and examine whether RRMS patients with increased immune activation show a higher relapse rate or an earlier relapse compared with RRMS patients without immune activation.

## 6. Conclusions

A considerable proportion of RRMS patients in remission exhibit active immune-inflammatory pathways and neuroimmune toxicity. Activated immune-inflammatory responses and an aberrant erythron may explain the presence of chronic fatigue, affective symptoms, physiosomatic symptoms, insomnia, and autonomic symptoms throughout the remission period of RRMS. MS-related chronic fatigue and affective symptoms are caused by immunological activation and erythrocyte abnormalities. The more generalized IRS and CIRS activation, as well as the Th1 profile and Th17-axis, are therapeutic targets to prevent subsequent relapses in a subset of remitted RRMS patients. Erythron abnormalities are emerging therapeutic targets for the treatment of RRMS-related chronic fatigue, and physiosomatic and mood symptoms.

## Figures and Tables

**Table 1:** Sociodemographic and blood parameters of multiple sclerosis (MS) patients and healthy controls (HC).

Variables	HC (n = 30)	MS Patients (n = 63)	F/X^2^	df	*p*-Value
Age (Years)	31.70(5.38)	29.76(8.40)	1.33	1/91	0.252
Sex (M/F)	19/11	23/40	5.90	1	0.015
Marital status (Married/Single)	19/11	29/34	2.43	1	0.119
Employment (Y/N)	28/2	11/52	48.04	1	<0.001
Smoking (Y/N)	5/25	9/54	0.090	1	0.764
BMI (Kg/m^2^)	26.57(4.53)	24.86(3.56)	3.92	1/91	0.051
Duration of illness (years)	-	5.51(4.72)	-	-	
MSSS	0	2.08 (2.01)	MWU	-	<0.001
EDSS	0	1 (1–2.5)	MWU	-	<0.001
ADL	14.00	13.06(1.40)	MWU	-	<0.001
PC_disabilities	−0.967(0)	0.518(0.659)	151.41	1/91	<0.001
Relapsing remission/Progressive MS	-	55/8	-	-	-
WBC (thousand/mm^3^)	6.59(1.75)	9.49(2.96)	24.59	1/91	0.009
RBC (million/mm^3^) *	4.915(0.111)	4.926(0.086)	0.00	1/81	0.940
Hct (%) *	42.974(0.947)	40.838(0.732)	3.08	1/81	0.083
Hb (g/dL) *	14.702(0.337)	14.175(0.261)	1.48	1/81	0.227
MCV (fl) *	87.931(1.249)	84.144(0.965)	5.58	1/81	0.021
MCH (pg) *	30.147(0.525)	28.269(0.405)	6.20	1/81	0.015
RDW-SD (fl) *	36.943(0.955)	41.322(0.738)	12.73	1/81	<0.001
PC_RBCs *	0.189(0.153)	−0.041(0.118)	1.37	1/81	0.245
PC_RBCindices *	0.332(0.183)	−0.084(0.141)	3.15	1/81	0.080
Pure depression	−0.964(1.002)	0.459(0.592)	73.62	1/91	<0.0013 **
Pure anxiety	−0.807(0.645)	0.384(0.907)	41.57	1/91	<0.0013 **
Pure physiosomatic	−1.223(0.524)	0.585(0.524)	243.61	1/91	<0.0013 **
Chronic fatigue	−0.912(0.578)	0.434(0.856)	60.68	1/91	<0.0013 **
Autonomic symptoms	−0.944(0.518)	0.449(0.849)	68.49	1/91	<0.0013 **
Insomnia	−0.824(0.532)	0.440(0.860)	63.54	1/91	<0.0013 **
PC_psychopathology	−1.228(0.445)	0.585(0.561)	240.69	1/91	<0.0013 **

All results (except EDSS, median, min-max) are shown as mean (SD); F/X^2^: results of analysis of variance and contingency tables, respectively; MWU: Mann–Whitney U (MWU) test; * Results of factorial GLM analysis with sex as a second factor (shown as mean and standard error); ** Results of false discovery rate *p*-correction. M: Male, F: Female, Y: Yes, N: No, BMI: Body Mass Index, Kg: Kilogram, m^2^: Meters squared, MSSS: Multiple Sclerosis Severity Scale, EDSS#: Expanded Disability Status Scale, ADL: Activities of daily living, Hb: Hemoglobin, Hct: Hematocrit, RBC: Red blood cells, MCV: Mean corpuscular volume, MCH: Mean corpuscular hemoglobin, RDW-SD: Red cell distribution width, WBC: White blood cells, dl: deciliter, g: gram, pg: picogram, fl: femtoliters, mm^3^: Cubic millimeter. PC_disabilties: principal component extracted from ADL, MSSS, and EDSS scores. PC_RBCs: principal component extracted from RBCs, Hct, and Hb. PC_RBCindices: principal component extracted from MCV, MCH, and RDW.

**Table 2:** Cytokine profiles of healthy controls (HC) and patients with multiple sclerosis (MS) divided into patients with increased (HIGH) and normal (LOW) immune activation.

Variables	HC (n = 30) ^A^	LOW MS (n = 34) ^B^	HIGH MS (n = 29) ^C^	F	dfh/dfe	*p*-Value
M1 (z scores) *	−0.347(0.079) ^C^	−0.210(0.077) ^C^	0.605(0.174) ^A,B^	19.43	2/90	0.002
Th1 (z scores) *	−0.329(0.096) ^C^	−0.159(0.130) ^C^	0.528(0.119) ^A,B^	12.42	2/90	0.002
Th2 (z scores)	−0.164(0.050) ^B^	0.135(0.062) ^A^	0.010(0.099)	5.72	2/90	0.018
zTh2-zTh1 (z scores)	−0.172(0.127)	0.175(0.187)	−0.027(0.211)	0.986	2/90	0.377
Th17 (z scores) *	−0.304(0.092) ^C^	−0.062(0.099) ^C^	0.388(0.186) ^A,B^	4.54	2/90	0.002
Th17-axis (z scores) *	−0.909(0.218) ^C^	−0.226(0.262) ^C^	1.20(0.443) ^A,B^	8.21	2/90	0.002
IRS (z scores) *	−0.308(0.079) ^C^	−0.212(0.090) ^C^	0.567(0.143) ^A,B^	20.35	2/90	0.002
T-cell growth (z scores) *	−0.147(0.070) ^C^	−0.216(0.076) ^C^	0.406(0.168) ^A,B^	9.08	2/90	0.002
Growth factors (z scores) *	−0.333(0.068) ^C^	−0.243(0.103) ^C^	0.629(0.190) ^A,B^	12.90	2/90	0.002
Neurotoxicity (z scores) *	−0.330(0.086) ^C^	−0.141(0.099) ^C^	0.507(0.136) ^A,B^	15.65	2/90	0.002
CIRS (z scores)	−0.225(0.076) ^B^	0.210(0.103) ^A^	−0.013(0.174)	3.26	2/90	0.044

Results of Quade’s nonparametric ANCOVA with age, sex, smoking, and body mass index as covariates and * with CIRS as an additional covariate; dfh = degrees of freedom for the hypothesis, dfe = degrees of freedom for error; data are expressed as mean (SE), i.e., estimated marginal means; ^A,B,C^: pairwise comparisons among group means. Shown are the false discovery rate corrected *p* values. M1: Macrophage M1, Th: T-helper, IRS: Immune inflammatory response, CIRS: Compensatory immune-regulatory system.

**Table 3:** Results of multiple regression analyses with disabilities and multiple sclerosis severity scale (MSSS) scores as dependent variables and different immune profiles as explanatory variables.

Dependent Variables	Explanatory Variables	Coefficients of Input Variables			Model Statistics			
		β	t	*p*	R^2^	F	df	*p*
In all subjects combined								
#1. PC_disabilities	Model				0.424	21.32	3/87	<0.001
	Th1	0.338	2.61	0.011				
	CIRS	0.269	3.27	0.002				
	Th17-axis	0.281	2.17	0.033				
#2. MSSS	Model				0.533	31.6	3/83	<0.001
	T-cell growth	0.252	1.72	0.089				
	CIRS	0.386	5.01	<0.001				
	Th1	0.445	3.01	0.004				
In patients only								
#3. MSSS	Model				0.178	6.06	2/56	0.004
	CIRS	0.397	3.04	0.004				
	Th1	0.356	2.72	0.009				

Th: T-helper, CIRS: Compensatory immune-regulatory system, MSSS: Multiple Sclerosis Severity Scale; PC_disabilities: principal component extracted from activities of daily living, multiple sclerosis severity scale, and expanded disability status scale.

**Table 4:** Results of multiple regression analyses with neuropsychiatric rating scale scores as dependent variables and immune profiles, erythron variables, and white blood cell (WBC) count as explanatory variables.

Dependent Variables	Explanatory Variables	Coefficients of Input Variables			Model Statistics			
		β	t	*p*	R^2^	F	df	*p*
#1. PC_PP (in all subjects)	Model				0.506	21.51	4/84	<0.001
	Th17-axis	0.425	5.1	<0.001				
	WBCs	0.304	3.6	<0.001				
	PC_RBCs	−0.269	−3.41	<0.001				
	CIRS	0.17	2.15	0.034				
#2. PC_PP (with forced entry of MSSS scale score)	Model				0.563	27.06	4/84	<0.001
	MSSS	0.425	4.98	<0.001				
	Sex	−0.228	−3.11	0.003				
	WBCs	0.247	3.19	0.002				
	T-cell growth	0.214	2.56	0.012				
#3. PC_PP (in patients only)	Model				0.171	5.98	4/58	0.004
	PC_RBCs	−0.358	−2.97	0.004				
	WBCs	0.254	2.11	0.039				
#4. Pure depression	Model				0.243	9.08	3/85	<0.001
	WBCs	0.324	3.19	0.002				
	PC_RBCs	−0.207	−2.15	0.035				
	Th17-axis	0.218	2.14	0.036				
#5. Pure anxiety	Model				0.347	15.08	3/85	<0.001
	Th17-axis	0.429	4.52	<0.001				
	WBCs	0.219	2.33	0.022				
	PC_RBCs	−0.189	−2.12	0.037				
#6. Pure physiosomatic	Model				0.503	21.21	4/84	<0.001
	Th17-axis	0.44	5.29	<0.001				
	WBCs	0.285	3.4	0.001				
	Sex	−0.225	−2.86	0.005				
	CIRS	0.174	2.19	0.032				
#7. Fatigue	Model				0.417	20.29	3/85	<0.001
	Neurotoxicity	0.45	5.06	<0.001				
	CIRS	0.301	3.55	<0.001				
	WBCs	0.186	2.05	0.043				
#8. Autonomic	Model				0.339	14.73	3/86	<0.001
	Neurotoxicity	0.364	3.85	<0.001				
	WBCs	0.301	3.19	0.002				
	PC_RBCs	−0.189	−2.12	0.037				
#9. Sleep	Model				0.33	13.97	3/85	<0.001
	IRS	0.359	3.83	<0.001				
	WBCs	0.264	2.85	0.005				
	Sex	−0.225	−2.50	0.014				

PC_PP: principal components computed from all symptom subdomains of the Hamilton depression rating scale (HAMD), Hamilton anxiety rating scale (HAMA), Fibro-Fatigue (FF) scale. Th: T-helper, PC_disabilities: principal component extracted from red blood cell number, hematocrit, and hemoglobin; WBCs: white blood cells count, CIRS: Compensatory immune-regulatory system, MSSS: Multiple sclerosis severity scale.

**Table 5:** Results of automatic linear modeling analyses (best subsets with overfit prevention criterion) with multiple sclerosis disabilities and psychopathology (PP) rating scales and subdomain scores as dependent variables and serum erythron variables, white blood cells count (WBCs), cytokines, chemokines, and growth factors as explanatory variables.

Dependent Variables	Explanatory	Coefficients of Input Variables					Corrected Model		
	Variables						Statistics		
		β	t	*p*	95%CI	Importance	F	Df	*p*
#1. PC_disabilities	Model						27.01	3/89	<0.001
	IL-17	0.321	3.59	0.001	0.143; 0.498	0.426			
	IFN-γ	0.324	3.24	0.002	0.126; 0.522	0.349			
	sIL-1RA	0.27	2.61	0.011	0.055; 0.405	0.225			
#2. PC_PP	Model						8.2	11/77	<0.001
	WBCs	0.328	3.79	<0.001 0.001	0.156; 0.501	0.313			
	PC_RBCs	−0.291	−3.61	0.011	−0.452; −0.131	0.283			
	IL-10	0.255	2.62	0.022	0.061; 0.488	0.149			
	IL-6	0.262	2.33		0.038; 0.485	0.118			
#3. Pure depression	Model						3.31	15/74	<0.001
	WBCs	0.43	3.98	<0.001	0.215; 0.645	0.322			
	PC_RBCs	−0.236	−2.44	0.017	−0.428; −0.043	0.121			
	IL-9	0.548	2.02	0.047	0.007; 1.089	0.083			
#4. Pure anxiety	Model						5.37	9/79	<0.001
	TNF-α	0.503	2.87	0.005	0.153; 0.852	0.429			
#5. Pure physiosomatic	Model						8.84	9/73	<0.001
	WBCs	0.114	4.05	<0.001	0.058; 0.171	0.475			
	PC_RBCs	−0.219	−2.58	0.012	−0.388; −0.050	0.193			
	IL-13	0.216	2.01	0.048	0.002; 0.431	0.117			
#6. Chronic fatigue	Model						5.94	13/75	<0.001
	IL-4	0.319	3.37	0.001	0.130; 0.508	0.323			
	IFN-γ	0.401	2.6	0.011	0.094–0.708	0.193			
#7. Autonomic	Model						4.49	12/77	<0.001
	MIP1A	0.438	2.53	0.014	0.093; 0.783	0.221			
	MCP1	0.279	2.38	0.02	0.045; 0.513	0.196			
#8. Sleep	Model						16.09	3/85	<0.001
	IL-13	0.397	4.77	0	0.232; 0.563	0.496			
	WBCs	0.352	3.95	0	0.175; 0.529	0.34			
	PC_RBCs	−0.244	−2.74	0.007	−0.422; −0.067	0.164			
#9. PC_PP (in patients only)	Model						3.6	12/48	<0.001
	PC_RBCs	−0.284	−3.48	<0.001	−0.448; −0.120	0.304			
	IL-10	0.191	2.85	0.001	−0.056; 0.325	0.204			
	IL-1Ra	−0.278	−2.51	0.006	−0.501; −0.055	0.158			

IFN-γ: Interferon-gamma, WBCs: White blood cells count, PC_disabilities: principal component extracted from three disability scores; PC_RBCs: principal component extracted from red blood cells, hematocrit, and hemoglobin, IL: Interleukin, TNF-α: Tumor necrosis factor-alpha, MIP1A: Macrophage Inflammatory Protein-1A: MCP1: Monocyte chemoattractant protein-1, IL-1RA: Interleukin receptor antagonist.

## Data Availability

After the authors have thoroughly utilized the dataset, the corresponding author (M.M.) will make all pertinent data available upon reasonable request.
